# Synergistic reduction in albuminuria in type 2 diabetic mice by esaxerenone (CS-3150), a novel nonsteroidal selective mineralocorticoid receptor blocker, combined with an angiotensin II receptor blocker

**DOI:** 10.1038/s41440-020-0495-0

**Published:** 2020-07-02

**Authors:** Kiyoshi Arai, Yuka Morikawa, Naoko Ubukata, Kotaro Sugimoto

**Affiliations:** 1 End-Organ Disease Laboratories, Daiichi Sankyo Co., Ltd., Tokyo, Japan; 2 Rare Disease & LCM Laboratories, Daiichi Sankyo Co., Ltd., Tokyo, Japan; 3 Medical Science Department, Daiichi Sankyo Co., Ltd., Tokyo, Japan; 4 Present Address: Global Project Management Department, Daiichi Sankyo Co., Ltd., Tokyo, Japan; 5 Present Address: Specialty Medicine Research Laboratories I, Daiichi Sankyo Co., Ltd., Tokyo, Japan

**Keywords:** Albuminuria, Angiotensin II receptor blocker, Diabetic nephropathy, Esaxerenone, Mineralocorticoid receptor blocker

## Abstract

Esaxerenone is a novel selective mineralocorticoid receptor (MR) blocker that was recently approved in Japan to treat hypertension. In phase II and III studies, esaxerenone plus a renin–angiotensin system inhibitor markedly reduced the urinary albumin-to-creatinine ratio (UACR) in hypertensive patients with diabetic nephropathy. To evaluate a direct renoprotective effect by MR blockade independent of an antihypertensive effect in the context of diabetic nephropathy, esaxerenone (3 mg/kg), olmesartan (an angiotensin II receptor blocker; 1 mg/kg), or both were orally administered to KK-Ay mice, a type 2 diabetes model, once daily for 56 days. Urinary albumin (Ualb), UACR, and markers, such as podocalyxin, monocyte chemoattractant protein-1 (MCP-1), and 8-hydroxy-2′-deoxyguanosine (8-OHdG), were measured, along with systolic blood pressure (SBP), fasting blood glucose, and serum K^+^ levels. Prior to the initiation of drug administration, KK-Ay mice showed higher blood glucose, insulin, Ualb excretion, and UACR levels than C57BL/6 J mice, a nondiabetic control, indicating the development of diabetic renal injury. Combined treatment with esaxerenone and olmesartan significantly reduced the change in UACR from baseline compared with the change associated with vehicle at week 8 (−1.750 vs. 0.339 g/gCre; *P* < 0.002) and significantly inhibited the change in Ualb from baseline compared with the change associated with vehicle at week 8 (*P* < 0.002). The combination treatment also reduced urinary excretion of podocalyxin and MCP-1, but did not influence 8-OHdG excretion, SBP, blood glucose, or serum K^+^ levels. Overall, esaxerenone plus olmesartan treatment ameliorated diabetic nephropathy in KK-Ay mice without affecting SBP, suggesting that the renoprotective effects of esaxerenone could be exerted independently of its antihypertensive effect.

## Introduction

Diabetic nephropathy is a serious complication that affects up to 40% of patients with type 1 or type 2 diabetes [1]. Diabetic nephropathy is one of the three most common diseases underlying the need for renal dialysis [2]; thus, preventing the progression of diabetic nephropathy could prevent worsening of quality of life [3]. One of the major causes of diabetic nephropathy progression is hypertension, and reducing blood pressure can be expected to slow the rate of advancement of renal impairment [4]. Since renin–angiotensin system (RAS) inhibitors are thought to have both antihypertensive and renoprotective activity, they are currently recommended in Japan [5] and elsewhere [6] as a first-line therapy for hypertensive patients with diabetic nephropathy.

Esaxerenone (CS-3150, MINNEBRO^®^, Daiichi Sankyo Co., Ltd.) is a novel selective mineralocorticoid receptor (MR) blocker with a nonsteroidal structure [7]. Esaxerenone has been shown to be more potent (IC_50_ 3.7 nM) in blocking the transcriptional activation of human MR than the steroidal MR blockers spironolactone (IC_50_ 66 nM) and eplerenone (IC_50_ 970 nM) [7]. Moreover, esaxerenone has no agonistic effect on the MR and no antagonistic or agonistic effect on the glucocorticoid, androgen, or progesterone receptors, unlike eplerenone and spironolactone [7]. Esaxerenone also has a longer half-life and more pronounced antihypertensive activity in rodents than steroidal MR blockers [7–10].

Esaxerenone was approved in Japan in January 2019 for the treatment of hypertension [11]. In a double-blind, randomized, phase III study of 1001 Japanese patients with essential hypertension (the ESAX-HTN study), esaxerenone 2.5 mg/day was demonstrated to have antihypertensive activity at least equivalent to that of the MR blocker eplerenone at a dosage of 50 mg/day, and was generally well tolerated [12]. A long-term (52 weeks) phase III study of esaxerenone, alone or in combination with a calcium channel blocker, or RAS inhibitor in Japanese patients with essential hypertension, indicated that esaxerenone plus an RAS inhibitor was able to significantly reduce systolic blood pressure (SBP)/diastolic blood pressure (DBP) from baseline, and no obvious safety concerns were identified after 52 weeks of treatment [13].

In patients with hypertension and diabetic nephropathy, it has recently been suggested that combination treatment comprising an RAS inhibitor plus an MR blocker can further improve renal outcomes compared with the effects of an RAS inhibitor alone [14]. The combination of eplerenone plus an RAS inhibitor has been shown to reduce the urinary albumin-to-creatinine ratio (UACR) at 52 weeks relative to the baseline level in nondiabetic hypertensive patients [15]. In two placebo-controlled clinical studies in diabetic patients, the MR blocker finerenone combined with an RAS inhibitor was shown to reduce albuminuria or UACR [16, 17]. In a phase II study of esaxerenone added to ongoing RAS inhibitor treatment in hypertensive or normotensive patients with type 2 diabetes and microalbuminuria, the UACR was dose dependently (1.25, 2.5, and 5 mg/day) reduced at the end of the treatment compared with the UACR level associated with placebo [18]. Similar results were confirmed in a phase III study, in which 12 weeks of treatment with low-dosage esaxerenone (titration from 1.25 to 5 mg/day) in combination with an RAS inhibitor was able to reduce both SBP and DBP, and decrease the UACR by 32.4% from baseline in hypertensive patients with type 2 diabetes and albuminuria [19].

Renoprotective effects of esaxerenone, including albuminuria reduction, have also been demonstrated in various animal models. In hypertensive rodent models, esaxerenone was shown to prevent renal injury [10, 20, 21], and in high-salt-treated type 2 diabetic KK-Ay mice, a model of human hypertensive diabetic nephropathy [22], esaxerenone exhibited renoprotective effects, including a reduction in renal inflammation and oxidative stress [23]. However, these models were all salt-loaded, and the renoprotective activity of esaxerenone was achieved simultaneously with its antihypertensive effects [10, 20, 21, 23]. Thus, one important topic remains to be explored: whether the renoprotective effects observed in rodent models on a high-salt diet are also exhibited in mice that are not given excessive salt, i.e., if the renoprotective action of esaxerenone is independent of its antihypertensive effect. Therefore, the objective of this study was to evaluate the antihypertensive-independent renoprotective effects of esaxerenone in KK-Ay mice, receiving a normal diet (a model without increased blood pressure), in combination with the RAS inhibitor olmesartan.

## Methods

### Animals

This study was conducted in compliance with the Act on Welfare and Management of Animals and all applicable standards for the care, storage and euthanasia of laboratory animals. The animal experiment protocol was approved by the Institutional Animal Care and Use Committee of the New Drug Research Center Inc. (Hokkaido, Japan; Deliberation No. 131030A).

Male mice (C57BL/6 J normal mice and KK-Ay mice) were obtained from CLEA Japan Inc. (Tokyo, Japan) at 6 weeks of age. Animals were housed at 22 ± 3 °C with 50 ± 20% humidity and a 12 h/12 h light/dark cycle. Feed was provided *ad libitum* throughout the study, except during fasting; water was provided *ad libitum* at all times. Animals aged 8 weeks with no observable abnormalities in their general condition were selected. When the animals were between 8 and 9 weeks of age, the following parameters were measured: body weight, 24-h urine collection (urinary albumin [Ualb] concentration, urine creatinine concentration, and urine output), SBP and heart rate, 4-h fasting blood glucose concentration and insulin concentration. Animals were grouped such that the baseline values were uniform among treatment groups.

### Experimental protocols

Esaxerenone (Daiichi Sankyo Co., Ltd.) and olmesartan (Daiichi Sankyo Co., Ltd.) were suspended in 0.5% methylcellulose-400 solution (0.5% MC) to obtain concentrations of 0.3 mg/mL and 0.1 mg/mL, respectively. These suspensions were then used for drug administration. Esaxerenone (3 mg/kg of mouse body weight), olmesartan (1 mg/kg of mouse body weight), both, or vehicle (0.5% MC) were orally administered once daily from 10 weeks of age for 8 weeks (56 consecutive days) using a syringe tube (Terumo Corp., Tokyo, Japan). C57BL/6 J normal mice (*n* = 8) were used as a nondiabetic control group, in which vehicle was administered. KK-Ay mice were treated with vehicle (*n* = 12), esaxerenone (*n* = 12), olmesartan (*n* = 12), or esaxerenone plus olmesartan (combination; *n* = 12). The researcher was blinded to drug administration. Treated KK-Ay mice were excluded from the analysis if their plasma glucose concentration was below the mean plasma glucose concentration of the C57BL/6 J mice, as such treated KK-Ay mice would not be suitable for use as a model for type 2 diabetes.

Body weight was measured twice weekly, and SBP was measured at 3 and 7 weeks after the initiation of drug administration. Blood pressure was measured (using a small animal nonpreheating noninvasive sphygmomanometer; MK-2000, Muromachi Kikai Co., Ltd., Tokyo, Japan) in a stable state five times for one individual, and the average value of three sets of data (excluding the highest and lowest values) was used.

### Sample collection

Every 4 weeks, 24-h urine samples were collected, and urinary parameters, i.e., albumin, creatinine, podocalyxin, monocyte chemoattractant protein-1 (MCP-1), and 8-hydroxy-2′-deoxyguanosine (8-OHdG), were measured. Animals were placed in a mouse metabolism cage (TOYORIKO Co. Ltd., Tokyo, Japan), and urine was collected for 24 h. The collected urine volume was measured, and centrifugation was performed at room temperature at 3000 r.p.m. for 10 min. Measurements were performed on the supernatant with a microplate reader (SpectraMax Plus 384; Molecular Devices, LLC., San Jose, CA, USA) using enzyme-linked immunosorbent assay (ELISA) kits according to the manufacturers’ instructions (podocalyxin: Exocell Inc, Philadelphia, PA, USA; MCP-1: R&D Systems Inc., Minneapolis, MN, USA; and 8-OHdG: Japan Institute for the Control of Aging, NIKKEN SEIL Co., Ltd., Shizuoka, Japan).

Ualb concentration was measured using the LBIS^®^ Mouse Albumin ELISA Kit (FUJIFILM Wako Shibayagi Corporation [formerly Shibayagi Corporation], Gunma, Japan), and creatinine concentration was measured using the Creatinine (urinary) Colorimetric Assay kit (Cayman Chemical Co., Ltd., Ann Arbor, MI, USA).

Blood glucose levels were measured at 3 and 7 weeks after the initiation of drug administration, and serum K^+^ levels were measured at necropsy. Capillary blood sampling from the tail vein was performed after 4 h of fasting. Approximately 100 μL of whole blood was collected per animal; each sample was subjected to centrifugation at 4 °C at 3000 r.p.m. for 15 min, followed by plasma collection. Blood glucose and insulin concentrations were measured using commercial kits according to the manufacturers’ instructions (blood glucose: FUJIFILM Wako Pure Chemical Corporation [formerly Wako Pure Chemical Industries, Ltd.], Osaka, Japan; and insulin: Morinaga Institute of Biological Science, Inc., Yokohama, Japan). The day after the last dose was administered, animals were weighed, and blood was collected from the abdominal aorta under anesthesia. The blood was left to stand at room temperature for 10–15 min and then centrifuged at 4 °C at 3000 r.p.m. for 15 min. The resulting sera were used to measure blood electrolytes (Na, K, and Cl) using a Hitachi 7070 automated chemistry analyzer (Hitachi Co., Ltd., Tokyo, Japan).

### Statistical methods

No formal sample size calculations or power analyses were conducted. Changes from baseline (∆Ualb and ∆UACR) were calculated. The area under the curve (AUC) from the baseline value to week 8 of administration was calculated according to the following equation:

$$\left( {\left[ {{\mathrm{C}}0 + {\mathrm{C4}}} \right] \times \left[ {4 - 0} \right]} \right)/2 + \left( {\left[ {{\mathrm{C}}4 + {\mathrm{C}}8} \right] \times \left[ {8 - 4} \right]} \right)/2,$$

where C*x* is the measurement at week *x*, and C0 is the baseline value.

Urinary excretions (ng/day) were calculated as urinary substance level (ng/mL) × urine volume (mL/day). Urinary podocalyxin, MCP-1, and 8-OHdG excretion in each group are expressed as the mean ± standard error of the mean. For samples with urinary MCP-1 levels below the detection limit (2 pg/mL), the measured values were imputed as 2 pg/mL. Urinary excretion values were compared between groups. First, an equal variance test (significance level 5%) for comparison between the control and vehicle groups was performed by *F*-test; Student’s *t*-test was performed when equal variance was assumed, and Aspin–Welch’s *t*-test was performed when unequal variance was assumed. The tests were two-sided with significance levels of 5% (*P* < 0.05) and 1% (*P* < 0.01). If a significant difference (either at the 5 or 1% level) was detected between the control and vehicle groups, further comparisons were made between the five groups (vehicle vs. esaxerenone, olmesartan, or combination; esaxerenone vs. combination; or olmesartan vs. combination) of KK-Ay mice using the Bonferroni correction to address the problem of multiple comparisons. For comparisons between the five groups, new significance levels of *P* < 0.01 and *P* < 0.002 were used (calculated as 0.05/5 = 0.01, and 0.01/5 = 0.002, respectively); a significant tendency was assumed for a comparison with a significance level of *P* < 0.02. No further statistical tests or normality analyses were performed.

SAS System Release 9.2 or higher (SAS Institute Inc., Cary, NC, USA) was used for statistical analysis.

## Results

### Development of diabetic nephropathy in KK-Ay mice

**Fig. 1:**
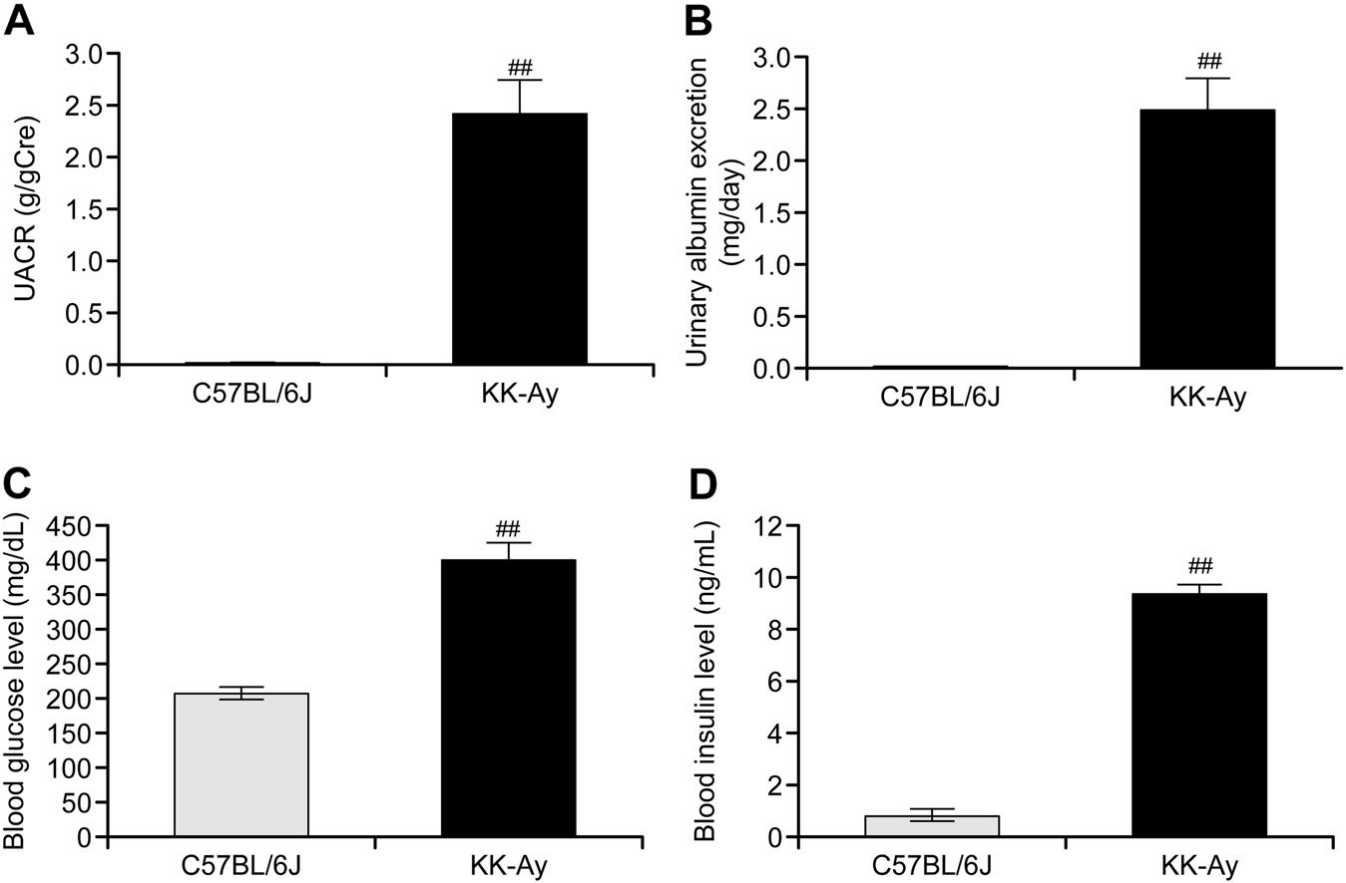
Urinary albumin-to-creatinine ratio (UACR) (**a**), urinary albumin excretion (Ualb) (**b**), fasting blood glucose (**c**), and insulin (**d**) in KK-Ay mice at 8 or 9 weeks of age. The data are given as the mean ± standard error. ^##^*P* < 0.01 vs. C57BL/6 J (comparison between two groups)

UACR, Ualb, blood glucose, and insulin levels were all increased significantly in KK-Ay mice compared with the respective levels in C57BL/6 J mice at 8–9 weeks of age (Fig. 1), indicating that KK-Ay mice developed diabetic nephropathy, as reported previously [22].

### Effects of esaxerenone and olmesartan combination treatment on UACR and Ualb

**Fig. 2:**
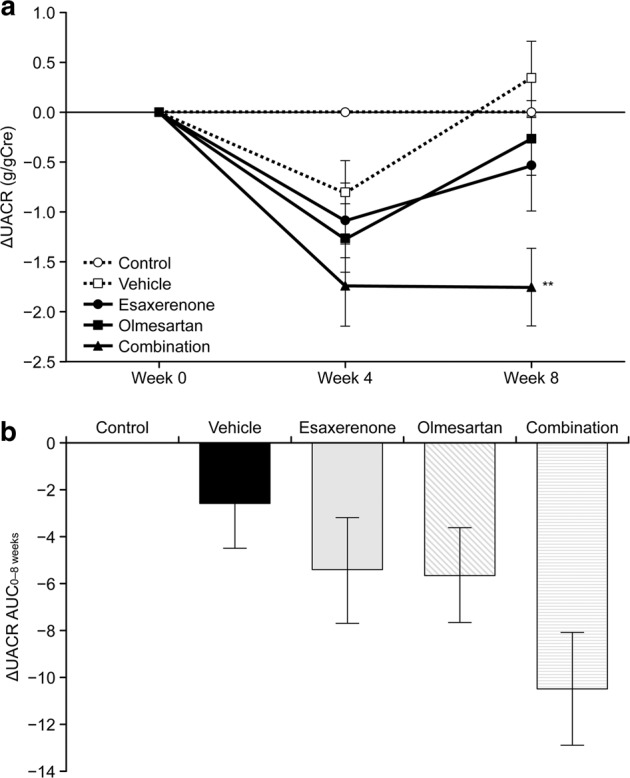
Effect of combined treatment with esaxerenone and olmesartan on the urinary albumin-to-creatinine ratio (UACR). **a** Time course of ∆UACR (change from baseline). **b** AUC of ∆UACR from 0 to 8 weeks. The data are given as the mean ± standard error. **P < 0.002 vs. vehicle (Bonferroni correction)

The effect of treatment with the combination of esaxerenone and olmesartan on UACR is shown in Fig. 2a. In the control group, UACR did not increase from baseline to week 8 (∆UACR at weeks 4 and 8 was 0.001 ± 0.001 and 0.002 ± 0.001 g/gCre, respectively), but increased in week 8 in the vehicle group (∆UACR at weeks 4 and 8 was −0.806 ± 0.315 and 0.339 ± 0.374 g/gCre, respectively). The trend toward increased ∆UACR at 8 weeks observed in the vehicle group was reduced in all other drug groups: ∆UACR at weeks 4 and 8 was −1.085 ± 0.366 and −0.526 ± 0.472 g/gCre, respectively, for the esaxerenone group; −1.269 ± 0.348 and −0.262 ± 0.374 g/gCre, respectively, for the olmesartan group; and −1.735 ± 0.410 and −1.750 ± 0.390 g/gCre, respectively, for the combination group (Fig. 2a). At week 8, the ∆UACR was significantly lower in the combination group than in the vehicle group using Bonferroni correction (−1.750 vs. 0.339 g/gCre, *P* < 0.002). Moreover, at week 8, ∆UACR was lower in the combination group than in the olmesartan group; this reduction was found to be significant when comparing the two groups (*P* = 0.012), but was not significant after implementing the Bonferroni correction. The AUC of ∆UACR from 0 to 8 weeks is shown in Fig. 2b. The difference in AUC between the combination group and the vehicle group was significant when comparing the two groups (*P* = 0.018), but was not significant after implementing the Bonferroni correction.

Ualb was significantly increased at weeks 4 and 8 in the vehicle group compared with the level in the control group (*P* < 0.01; Table 1). Combination treatment significantly decreased the Ualb change from baseline (∆Ualb) compared with the change observed in the vehicle group at week 8 (*P* < 0.002), as well as when compared with ∆Ualb associated with olmesartan at week 8 (*P* < 0.01). Esaxerenone alone tended to suppress ∆Ualb compared with the effect of vehicle at week 8 (*P* = 0.019), but the effect of olmesartan alone was not significantly different from that of vehicle.

**Table 1:** Effect of combination treatment with esaxerenone and olmesartan on urinary albumin excretion at weeks 4 and 8 compared with baseline

	Weeks	Control (*n* = 8)	Vehicle (*n* = 12)	Esaxerenone (*n* = 11)^a^	Olmesartan (*n* = 12)	Combination (*n* = 12)
Ualb^b^ (mg/day)	0	0.003 ± 0.000	2.490 ± 0.295^##^	2.378 ± 0.306	2.729 ± 0.303	2.330 ± 0.363
	4	0.006 ± 0.001	2.238 ± 0.382^##^	1.392 ± 0.252	1.996 ± 0.230	1.038 ± 0.113
	8	0.004 ± 0.001	3.823 ± 0.392^##^	1.978 ± 0.457	3.315 ± 0.441	1.094 ± 0.202
ΔUalb^c^ (Ualb change from baseline) (mg/day)	0	—	—	—	—	—
	4	0.003 ± 0.001	−0.251 ± 0.288	−0.986 ± 0.466	−0.733 ± 0.347	−1.292 ± 0.376
	8	0.002 ± 0.001	1.333 ± 0.380	−0.401 ± 0.575	0.586 ± 0.417	−1.236 ± 0.379**^,$^

The data are given as mean ± standard error

^##^*P*  <  0.01 vs. control (comparison between two groups); **P  <  0.002 vs. vehicle (Bonferroni correction); ^$^*P*  <  0.01 vs. olmesartan (Bonferroni correction)

^a^One animal met the criteria for exclusion and data were not included in the analysis

^b^For Ualb, the test for significant difference was conducted only between the control and vehicle groups

^**c**^For ΔUalb, the test for significant difference was conducted only for the following combinations: vehicle vs. esaxerenone, vehicle vs. olmesartan, vehicle vs. combination, esaxerenone vs. combination, and olmesartan vs. combination

### Effects of esaxerenone and olmesartan on blood and serum parameters

**Fig. 3:**
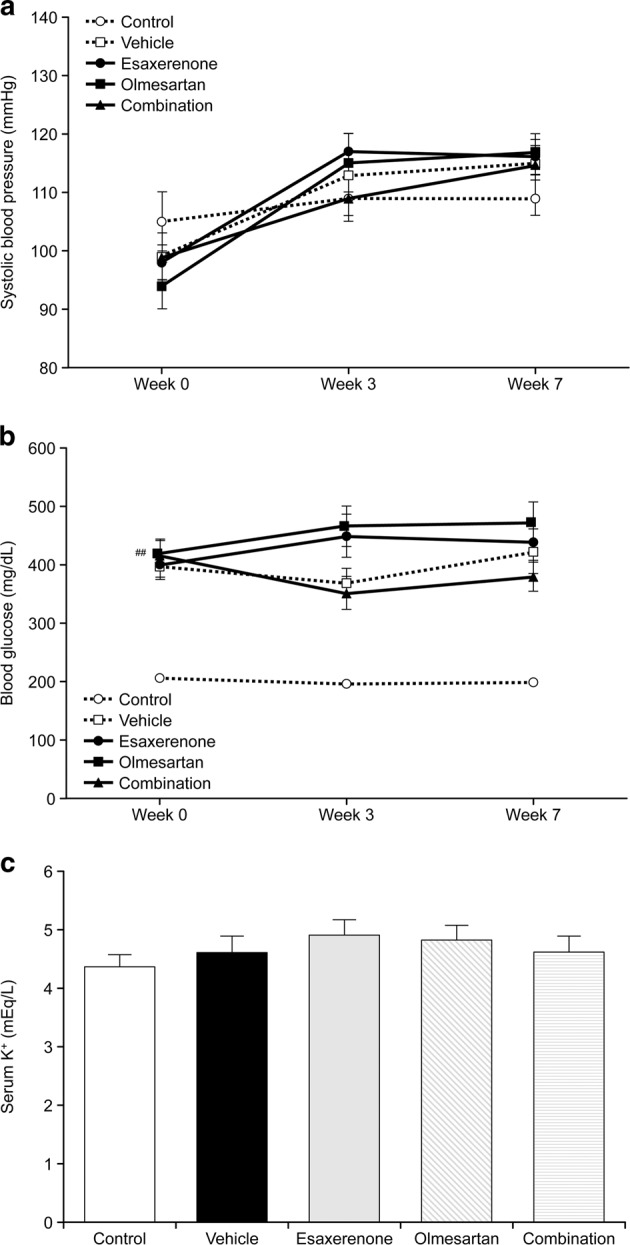
Effect of combination treatment with esaxerenone and olmesartan on systolic blood pressure (**a**), fasting blood glucose (**b**), and serum K^+^ level (**c**). Neither esaxerenone, olmesartan, nor combination treatment had any significant effect on systolic blood pressure, fasting blood glucose, or serum K^+^ levels. The data are given as the mean ± standard error. ^##^*P* < 0.01 vehicle vs. control (comparison between two groups)

Neither esaxerenone, olmesartan, nor combination treatment had any significant effect on SBP, fasting blood glucose, or serum K^+^ levels (Fig. 3a–c).

### Effects of esaxerenone and olmesartan combination treatment on podocalyxin, MCP-1, and 8-OHdG

The combination treatment reduced urinary excretion of podocalyxin (biomarker of podocyte injury) and MCP-1 (inflammation marker), but did not influence 8-OHdG excretion (Supplementary Table 1).

**Fig. 4:**
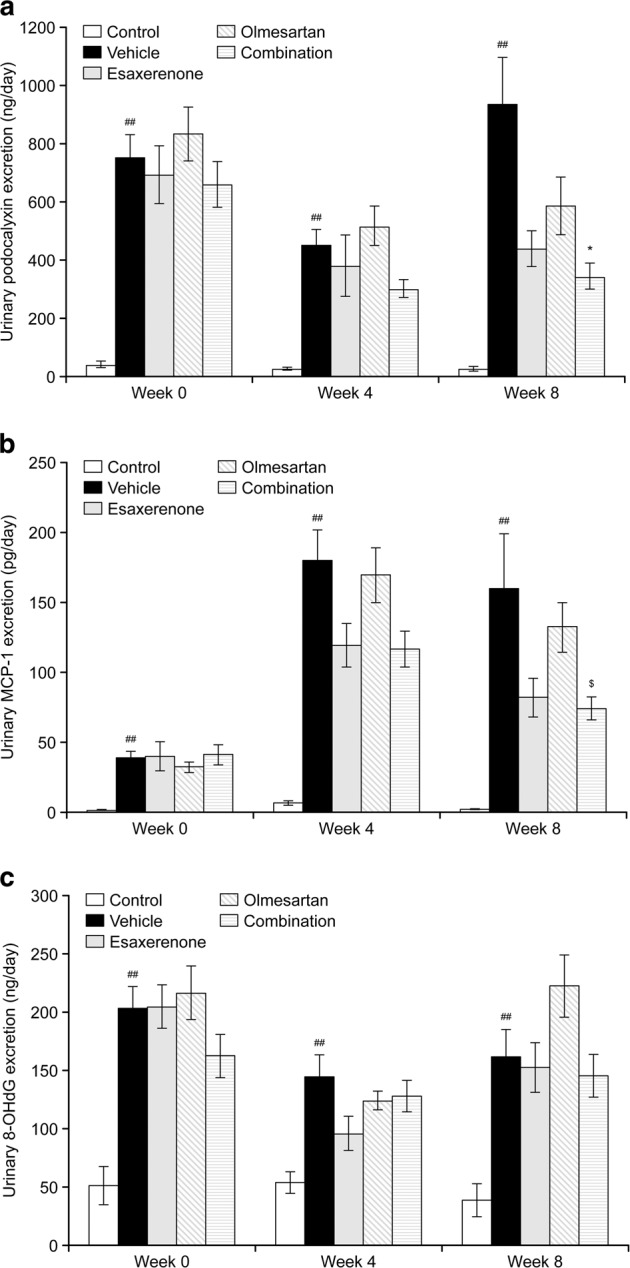
Effects of combination treatment with esaxerenone and olmesartan on urinary podocalyxin (**a**), MCP-1 (**b**), and 8-OHdG (**c**) excretion from 0 to 8 weeks in KK-Ay mice. Esaxerenone, olmesartan, or both were orally administered to KK-Ay mice once a day for 8 weeks. Twenty-four-hour urine collection was performed in weeks 0, 4, and 8. Urinary concentrations of each substance were measured, and urinary excretion for 24 h was calculated. The data are given as the mean ± standard error. MCP-1 monocyte chemoattractant protein-1, 8-OHdG 8-hydroxy-2’-deoxyguanosine. ^##^*P* < 0.01 vs. control (comparison between two groups); *P < 0.01 vs. vehicle (Bonferroni correction); ^$^*P* < 0.01 vs. olmesartan (Bonferroni correction)

Compared with the vehicle, the combination treatment tended to suppress urinary podocalyxin excretion at weeks 4 and 8 (week 4: *P* = 0.018, *n* = 12; week 8: *P* < 0.01, *n* = 12; Fig. 4a). Esaxerenone alone also suppressed urinary podocalyxin excretion at 8 weeks compared with the urinary podocalyxin excretion associated with the vehicle; this was significant when comparing the two groups (*P* = 0.011, *n* = 11), but was not significant after performing the Bonferroni correction. Olmesartan had no significant effect. The combination treatment showed a tendency to suppress urinary podocalyxin excretion at 4 and 8 weeks of treatment compared with the effects of olmesartan; the reduction was significant when comparing the two groups (week 4: *P* = 0.011, *n* = 12; week 8: *P* = 0.04, *n* = 12), but the significance was not observed after performing Bonferroni correction.

Combination treatment resulted in lower urinary MCP-1 excretion than the vehicle group; this treatment group exhibited a significant tendency toward reduction at week 4 (*P* = 0.0199, *n* = 12), but not at week 8 (*P* = 0.051, *n* = 12; Fig. 4b). However, although not statistically significant, the week 8 data suggested a continued trend toward decreased MCP-1 excretion with combination treatment. Compared with the vehicle group, esaxerenone monotherapy showed a nonsignificant trend toward a decrease in MCP-1 excretion at 4 (*P* = 0.036, *n* = 11) and 8 weeks (*P* = 0.078, *n* = 11); however, there were no specific effects with olmesartan alone. Combination treatment significantly suppressed urinary MCP-1 excretion at week 8 of treatment compared with the effect of olmesartan (*P* < 0.01, *n* = 12).

There was no significant effect on urinary 8-OHdG excretion compared with the effect of the vehicle when the animals were administered esaxerenone alone, olmesartan alone, or the combination treatment (Fig. 4c).

### Effects of esaxerenone and olmesartan on body weight and organ size

Compared with the control group of C57BL/6 J mice, vehicle-treated KK-Ay mice were significantly heavier during the entire period from 0 to 56 days (*P* < 0.01, comparison between two groups). Mice in the esaxerenone group showed a significant reduction in weight gain compared with the weight gain of the vehicle group from days 3 to 31 (*P* < 0.01, Bonferroni correction), but not from day 35 onward. The reduction in weight gain was not significant between the combination and vehicle groups after performing Bonferroni correction. However, when compared between two groups, the combination group exhibited a significant reduction in weight gain compared with the vehicle group (*P* < 0.05 for all days except days 1, 10, 52, and 57), as did the esaxerenone vs. vehicle groups (*P* < 0.05 for all days except day 52; Supplementary Fig. 1).

Neither active agent nor the combination treatment had any adverse impact on organ size compared with the impact of the vehicle. At week 8, the mean weight of the bilateral kidneys was 13.344 ± 0.252 mg/g body weight in the vehicle group, 14.034 ± 0.275 mg/g body weight in the esaxerenone group, 14.342 ± 0.343 mg/g body weight in the olmesartan group, and 13.435 ± 0.282 mg/g body weight in the combination group. The mean weights of the heart were 4.198 ± 0.149 mg/g body weight (vehicle), 4.172 ± 0.099 mg/g body weight (esaxerenone), 4.232 ± 0.088 mg/g body weight (olmesartan), and 3.910 ± 0.079 mg/g body weight (combination); the mean weights of the lung were 3.155 ± 0.060, 3.269 ± 0.083, 3.276 ± 0.065, and 3.464 ± 0.099 mg/g body weight, respectively.

## Discussion

There is currently considerable interest in the use of combination treatment with an RAS inhibitor plus MR blocker to treat hypertension and improve renal outcomes in patients with diabetes [14, 24], and whether the renoprotective effects of MR blockers are independent of changes in blood pressure. This study was designed to examine the renoprotective effects of esaxerenone plus olmesartan in nonhypertensive diabetic nephropathy model mice (KK-Ay mice [22, 25]) receiving a normal diet, i.e., without salt loading. As expected, the KK-Ay mice developed symptoms of diabetic nephropathy at 8−9 weeks of age, with significant increases in UACR, Ualb excretion, plasma glucose, and insulin concentrations compared with the respective outcomes in the control C57BL/6 J mice. When esaxerenone plus olmesartan was administered orally to the KK-Ay mice for 56 days, significant inhibition was observed in ∆Ualb and in ∆UACR at week 8 of administration compared with the respective outcomes of the vehicle group, independent of changes in blood pressure and glucose metabolism. Of note, when compared with olmesartan alone, the combination treatment showed a significant reduction in ∆Ualb; however, the combination treatment only showed a significant inhibition of ∆UACR when comparing the two groups (*P* = 0.04), but not when Bonferroni correction was used. Furthermore, esaxerenone plus olmesartan suppressed increases in urinary podocalyxin and MCP-1 levels. These results suggested that administration of esaxerenone plus olmesartan improved albuminuria by suppressing the progression of podocyte injury and inflammation in KK-Ay mice without salt loading.

To date, it has been unclear whether the renoprotective effect of MR blockers observed in clinical studies occurs as a direct result of blood pressure reduction [15–19]. Clinical data of eplerenone, spironolactone, and finerenone suggested the coexistence of both antihypertensive-dependent and antihypertensive-independent renoprotective effects of MR blockers [16, 17, 26]. The ARTS-DN study of finerenone 1.25–20 mg/day added to an RAS inhibitor in patients with diabetes and high or very high albuminuria demonstrated a dose-dependent reduction in UACR, but blood pressure was only modestly reduced, even at the highest dose [17]. In contrast, a second study of finerenone 1.25–20 mg/day in Japanese patients with type 2 diabetes mellitus and diabetic nephropathy suggested a correlation between changes in blood pressure and in UACR [16]. Other studies of steroidal MR blockers have also shown clear simultaneous reductions in blood pressure and albuminuria after 3 months of treatment [26].

Similarly, the results from nonclinical studies have also failed to clearly distinguish between the antihypertensive and renoprotective effects of MR blockers [10, 21, 27]. However, findings from several studies with esaxerenone have suggested that these two effects are independent. In a study of deoxycorticosterone acetate/salt-induced hypertensive rats, a clear amelioration of existing renal injury by esaxerenone was observed without SBP reduction, suggesting that the two activities (renoprotection and antihypertension) were independent [20]. In another study of salt-loaded KK-Ay mice, the antihypertensive effects of spironolactone and esaxerenone were equivalent, but the renoprotective effects of esaxerenone (attenuation of albuminuria, glomerular injury, tubulointerstitial fibrosis, and renal inflammation) were stronger [23], again suggesting that the renoprotective effects of esaxerenone may not depend on antihypertensive effects. In a series of previous animal studies examining the antihypertensive effects of MR blockers, including esaxerenone, the evaluation system used salt loading to increase blood pressure. In contrast, this study was conducted in a nonhypertensive model without salt loading and could investigate the renoprotective effect without being affected by elevated blood pressure.

It was suggested that the antihypertensive-independent renoprotective effect of esaxerenone was due to suppression of inflammation and the subsequent reduction in renal oxidative stress [20, 23]. In fact, several organ protection mechanisms of MR blockers have been reported in the literature, including inhibition of inflammation, podocyte injury, fibrosis, and oxidative stress [28–30]. The results of the current analysis are generally in agreement with these reports, although notably, administration of esaxerenone (alone or in combination) in our study did not suppress renal oxidative stress (urinary 8-OHdG level) despite the fact that combination treatment was able to decrease several other markers of renal injury, including albuminuria and biomarkers of inflammation. The reasons for this remain unclear, but we can hypothesize a possible explanation for this apparent discrepancy. We know that 8-OHdG is a marker for oxidative stress throughout the body [31] rather than a selective marker for renal injury. It is also well known that glucose metabolism is closely related to oxidative stress and that a high level of blood glucose increases oxidative stress [32]. In this study, KK-Ay mice had high levels of blood glucose and insulin, which may have induced systemic oxidative stress, and it is possible that KK-Ay mice developed damage to other organs in addition to the kidneys. Since such damage would likely be unaffected by administration of esaxerenone or olmesartan, 8-OHdG levels would remain unchanged in this case. This hypothesis is consistent with the result that esaxerenone or olmesartan did not affect blood glucose levels. Further research will be necessary to clarify the exact mechanisms underlying the renoprotective effects of esaxerenone and olmesartan. Nevertheless, regardless of the unchanged 8-OHdG levels in this study, combination treatment was shown to have impacts on other renal parameters, indicating its potential clinical utility in patients with diabetic nephropathy.

In this nonclinical study, the MR blocker esaxerenone demonstrated synergistic renoprotective effects in combination with an RAS inhibitor. There is accumulating preclinical [27] and clinical [14–17] evidence for the use of other MR blockers in combination with RAS inhibitors, suggesting that the addition of an MR blocker to an RAS inhibitor may provide additional benefits to patients beyond reduction in blood pressure. In fact, recent clinical studies have indicated that adding esaxerenone to an RAS inhibitor enhances both antihypertensive and antialbuminuric effects compared with the effects of an RAS inhibitor alone [13, 18, 19]. Moreover, as might be expected, vehicle-treated KK-Ay mice exhibited significant increases in body weight during this study compared with the body weight of the control group of C57BL/6 J mice, likely due to the diseased state. Both esaxerenone monotherapy and the combination treatment reduced this weight gain, with treated mice showing weight changes more in line with those observed in the control group, suggesting that combination therapy may have additional benefits beyond antihypertensive and renoprotective activities. However, similar effects on body weight remain to be confirmed in clinical studies.

Unfortunately, MR blockers have side effects, such as hyperkalemia [14, 33, 34], which limits their clinical usefulness in patients with diabetic nephropathy. Hyperkalemia is also a common adverse event associated with RAS inhibitors [35–37], and the addition of a steroidal MR blocker to an RAS inhibitor can significantly increase the risk of hyperkalemia (*P* < 0.00001) [38]. Thus, monitoring serum K^+^ levels is crucial in patients with nephropathy receiving both MR blockers and RAS inhibitors. Although hyperkalemia was not observed in this study, it is difficult to predict clinical outcomes from nonclinical experimental results; therefore, the results of two phase III clinical studies of esaxerenone in patients with diabetic nephropathy are in progress (JapicCTI-173695 and JapicCTI-173696).

Although liver weight and hepatic function were not evaluated in this study, esaxerenone and olmesartan are prescription drugs, and no hepatotoxicity concerns were identified in internal toxicity studies in mice (data on file, Daiichi Sankyo Co., Ltd.). In studies investigating the effects of esaxerenone administration in mice and rats for 3–6 months, hepatotoxicity of esaxerenone was not observed, and the no-observed-adverse-effect level was 30 mg/kg for mice (data on file, Daiichi Sankyo Co., Ltd). In a single-dose test in mice, the minimum lethal dose of olmesartan was 1700–1850 mg/kg for both sexes (data on file, Daiichi Sankyo Co., Ltd.). As the doses of esaxerenone and olmesartan used in our study were much lower than the toxic doses for the respective drugs, the risk of hepatotoxicity was considered to be extremely low.

Overall, the results of this study suggest that the combination of esaxerenone and olmesartan had a renoprotective effect in KK-Ay mice independent of the antihypertensive effect (without high-salt treatment). These data support and expand upon the results from a previous study in KK-Ay mice that were fed a high-salt diet, in which esaxerenone attenuated albuminuria, glomerular injury, tubulointerstitial fibrosis, and renal inflammation, which were in turn associated with reduced renal oxidative stress [23].

### Limitations

This study has several limitations. Data on renal injury were limited since observation of pathological sections of the kidney was not performed, and the expression of inflammation and oxidative stress markers in kidney sections was not evaluated. As discussed, data from a prior study in salt-loaded KK-Ay mice indicated that esaxerenone decreased albuminuria, inflammation, and renal injury through the suppression of oxidative stress [23], but based on the current data, the premise of whether esaxerenone reduces renal oxidative stress is a topic for further study. Future studies would also benefit from an examination into whether the suppressive effects of esaxerenone plus olmesartan on podocyte injury and renal inflammation are caused by inhibition of oxidative stress or by other mechanisms that have not yet been identified. In addition, as this was an exploratory study, no formal sample size calculations or power analyses were conducted, and the multiplicity of test results was not adjusted.

## Conclusions

Under the conditions of the present study, combined administration of esaxerenone and olmesartan inhibited diabetic nephropathy without affecting SBP in KK-Ay mice with spontaneous-onset diabetic nephropathy, suggesting that this treatment combination is able to exert renoprotective effects independently of the antihypertensive effect. The results from ongoing clinical studies of esaxerenone in diabetic nephropathy patients are eagerly awaited.

## Supplementary information

Supplementary Table 1

Supplementary Figure 1
