# Supplementary material for: Synergistic reduction in albuminuria in type 2 diabetic mice by esaxerenone (CS-3150), a novel nonsteroidal selective mineralocorticoid receptor blocker, combined with an angiotensin II receptor blocker
Source: Hypertens Res. 2020 Jul 2;43(11):1204–13. doi: 10.1038/s41440-020-0495-0 (PMC7685977; doi:10.1038/s41440-020-0495-0)
Supplement: Supplementary file 1 — Supplementary Table 1 [file 41440_2020_495_MOESM1_ESM.docx]

## Supplementary Table 1. Concentration levels and excretion of urinary biomarkers

| **Biomarkers** | **Weeks** | **Control  (n=8)** | **Vehicle  (n=12)** | **Esaxerenone (n=11)^2^** | **Olmesartan (n=12)** | **Combination (n=12)** |
| --- | --- | --- | --- | --- | --- | --- |
| Urinary volume (mL/day) | 0 | 0.4±0.1 | 7.9±0.8 | 7.8±0.6 | 8.3±0.8 | 7.5±0.8 |
|  | 4 | 0.7±0.1 | 9.4±1.4 | 7.7±1.6 | 11.2±0.8 | 8.4±1.0 |
|  | 8 | 0.6±0.1 | 11.6±1.3 | 9.1±1.6 | 12.8±1.1 | 9.1±1.5 |
| Urinary podocalyxin excretion (ng/day)^1^ | 0 | 40.6±10.4 | 754.9±76.4^##^ | 693.2±99.1 | 835.0±91.8 | 659.4±77.7 |
|  | 4 | 25.9±4.9 | 455.2±52.7^##^ | 382.0±105.3 | 516.4±67.8 | 300.2±30.2 |
|  | 8 | 26.5±8.3 | 938.1±157.7^##^ | 439.4±61.4 | 588.1±99.6 | 342.7±44.1* |
| Urinary MCP-1 excretion (pg/day)^1^ | 0 | 1.0±0.2 | 38.8±4.8^##^ | 40.2±10.6 | 32.1±3.7 | 41.0±7.1 |
|  | 4 | 6.6±1.4 | 180.2±21.8^##^ | 119.5±15.4 | 169.6±19.5 | 116.7±12.9 |
|  | 8 | 1.9±0.4 | 160.2±38.8^##^ | 81.9±13.8 | 132.2±17.8 | 74.3±8.2^$^ |
| Urinary 8-OHdG excretion (ng/day)^1^ | 0 | 50.9±16.3 | 203.4±18.3^##^ | 204.7±18.6 | 216.6±23.0 | 162.2±18.6 |
|  | 4 | 53.7±9.3 | 144.6±18.9^##^ | 95.8±14.8 | 124.0±7.9 | 127.7±13.5 |
|  | 8 | 38.5±13.9 | 161.4±23.6^##^ | 152.6±21.3 | 222.5±26.7 | 145.3±18.5 |

The data are given as mean ± standard error.

^1^Calculated by multiplying serum concentrations of each biomarker and urinary volume.

^2^One animal met the criteria for exclusion and data were not included in the analysis.

^##^P<0.01 vs. control (comparison between two groups).

*P<0.01 vs. vehicle, ^$^P<0.01 vs. olmesartan (Bonferroni correction).

8-OHdG, 8-hydroxy-2'-deoxyguanosine; MCP-1, monocyte chemoattractant protein-1
