# Supplementary figures and images for: Synergistic reduction in albuminuria in type 2 diabetic mice by esaxerenone (CS-3150), a novel nonsteroidal selective mineralocorticoid receptor blocker, combined with an angiotensin II receptor blocker
Source: Hypertens Res. 2020 Jul 2;43(11):1204–13. doi: 10.1038/s41440-020-0495-0 (PMC7685977; doi:10.1038/s41440-020-0495-0)

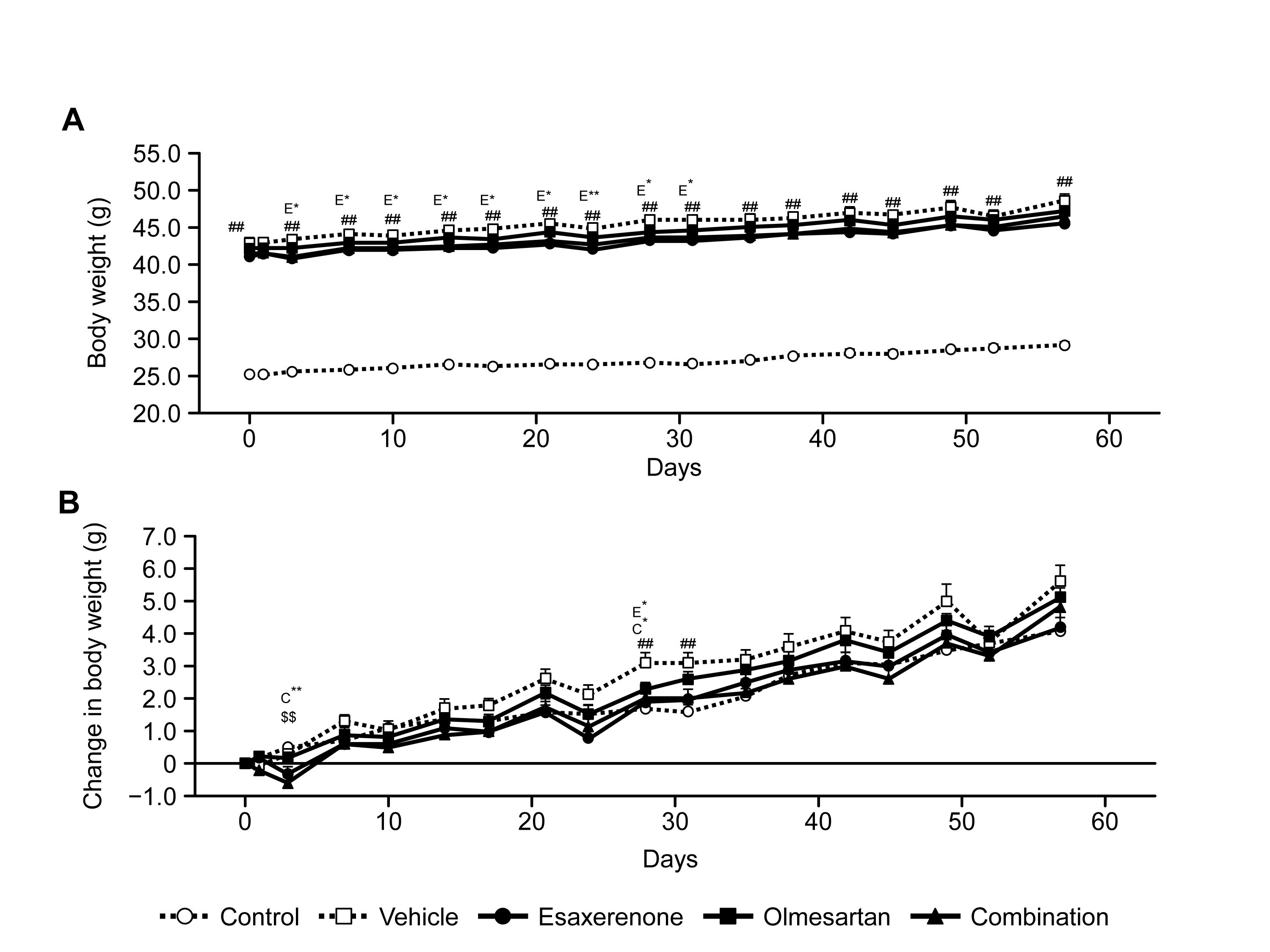

Supplement: Supplementary file 2 — Supplementary Figure 1 [file 41440_2020_495_MOESM2_ESM.tif]
